# Time to diagnosis of Type I or II invasive epithelial ovarian cancers: a multicentre observational study using patient questionnaire and primary care records

**DOI:** 10.1111/1471-0528.13447

**Published:** 2015-05-29

**Authors:** AWW Lim, D Mesher, A Gentry‐Maharaj, N Balogun, M Widschwendter, I Jacobs, P Sasieni, U Menon

**Affiliations:** ^1^ Centre for Cancer PreventionWolfson Institute of Preventive MedicineQueen Mary University of LondonBarts & The London School of Medicine and DentistryLondonUK; ^2^ Gynaecological Cancer Research CentreWomen's CancerInstitute for Women's HealthUniversity College LondonLondonUK; ^3^ Faculty of Medical and Human SciencesUniversity of ManchesterManchesterUK

**Keywords:** Delays, early diagnosis, ovarian cancer, symptoms, Type I and II epithelial ovarian cancer

## Abstract

**Objective:**

To compare time to diagnosis of the typically slow‐growing Type I (low‐grade serous, low‐grade endometrioid, mucinous, clear cell) and the more aggressive Type II (high‐grade serous, high‐grade endometrioid, undifferentiated, carcinosarcoma) invasive epithelial ovarian cancer (iEOC).

**Design:**

Multicentre observational study.

**Setting:**

Ten UK gynaecological oncology centres.

**Population:**

Women diagnosed with primary EOC between 2006 and 2008.

**Methods:**

Symptom data were collected before diagnosis using patient questionnaire and primary‐care records. We estimated patient interval (first symptom to presentation) using questionnaire data and diagnostic interval (presentation to diagnosis) using primary‐care records. We considered the impact of first symptom, referral and stage on intervals for Type I and Type II iEOC.

**Main outcome measures:**

Patient and diagnostic intervals.

**Results:**

In all, 78% of 60 Type I and 21% of 134 Type II iEOC were early‐stage. Intervals were comparable and independent of stage [e.g. median patient interval for Type I: early‐stage 0.3 months (interquartile range 0.3–3.0) versus late‐stage 0.3 months (interquartile range 0.3–4.5), *P *= 0.8]. Twenty‐seven percent of women with Type I and Type II had diagnostic intervals of at least 9 months. First symptom (questionnaire) was also similar, except for the infrequent abnormal bleeding (Type I 15% versus Type II 4%, *P *= 0.01). More women with Type I disease (57% versus 41%, *P *= 0.04) had been referred for suspected gynaecological cancer. Median time from referral to diagnosis was 1.4 months for women with iEOC referred via a 2‐week cancer referral to any specialty compared with 2.6 months (interquartile range 2.0–3.7) for women who were referred routinely to gynaecology.

**Conclusion:**

Overall, shorter diagnostic delays were seen when a cancer was suspected, even if the primary tumour site was not recognised to be ovarian. Despite differences in carcinogenesis and stage for Type I and Type II iEOC, time to diagnosis and symptoms were similar. Referral patterns were different, implying subtle symptom differences. If symptom‐based interventions are to impact on ovarian cancer survival, it is likely to be through reduced volume rather than stage‐shift. Further research on histological subtypes is needed.

**Tweetable abstract:**

No difference in time to diagnosis for Type I versus Type II invasive epithelial ovarian cancers.

## Tweetable abstract

No difference in time to diagnosis for Type I versus Type II invasive epithelial ovarian cancers.

## Introduction

Ovarian cancer remains the most lethal gynaecological cancer in the UK.^1^ There is an enduring perception that delays in presentation and onward referral are the root cause. Diagnosis relies on symptomatic presentation because population‐based screening has yet to show a mortality benefit.^2^ This has led to major efforts to shorten the time to diagnosis by raising public awareness of symptoms, encouraging women to present earlier, and issuing guidance encouraging primary‐care physicians to investigate women with a given symptom profile.^3^, ^4^, ^5^

Numerous studies^6^, ^7^, ^8^, ^9^, ^10^, ^11^, ^12^, ^13^, ^14^ have examined time to ovarian cancer diagnosis intervals, with median symptom onset to diagnosis varying from <1 month^8^ to >12 months.^7^, ^12^ Common to all the reports is the underlying premise that ovarian cancer is a single disease entity. This is at odds with our understanding that ovarian cancer is a heterogeneous disease, and that the key to improving outcomes is focusing on invasive epithelial ovarian cancer (iEOC), particularly the more aggressive high‐grade serous carcinomas.^15^ There is growing consensus that iEOC consists of five histological subtypes (low‐grade serous, clear cell, endometrioid, mucinous and high‐grade serous cancers), which differ in their underlying biology.

Invasive epithelial ovarian cancer can be grouped broadly into indolent Type I cancers (comprising low‐grade serous, clear cell, endometrioid, mucinous) that are often diagnosed at early stages with more favourable prognosis, and highly aggressive, rapidly evolving Type II tumours (high‐grade serous and its variants) that are typically diagnosed at advanced stages and are associated with poor survival.^16^, ^17^, ^18^ Type I tumours are thought to develop in a stepwise fashion from benign/borderline ovarian neoplasms,^19^, ^20^ whereas accumulating evidence suggests that Type II cancers arise from precursor lesions in the fallopian tubes.^18^ While type‐specific classification is likely to be an oversimplification of non high‐grade serous histotypes,^21^, ^22^ it broadly represents two groups of interest; aggressive, rapidly advancing tumours and slow‐growing tumours. We hypothesised that if time to diagnosis rather than biology was a major contributor to stage at diagnosis, the significant stage differences in the two iEOC subtypes should be accompanied by differences in symptoms and time to diagnosis. This is important because many symptom awareness efforts are underway with little evidence to suggest that a symptoms‐based tool could achieve earlier diagnosis.

This paper aims to provide the first estimates of time‐to‐diagnosis intervals for Type I versus Type II iEOC using two different data sources (patient questionnaires and primary‐care records) in a multicentre setting. We also compare intervals for borderline tumours versus iEOC.

### Methods

We obtained the relevant ethics approval from the Joint University College London/University College Hospital London Research Ethics Committee (05/Q0505/58). All participants provided written informed consent and were enrolled in the UK Ovarian Cancer Population Study (UKOPS—a biobank case–control study). Recruitment was from ten centres across England, Wales and Northern Ireland between February 2006 and February 2008. We only included women aged ≥45 years with primary EOC (International Classification of Diseases tenth revision, code C56) who were recruited before definitive diagnosis or treatment.

### Confirmation of diagnosis

An independent gynaecological oncologist confirmed the original diagnosis [morphology, grade and International Federation of Gynecology and Obstetrics (FIGO) stage] by review of pathology/cytology reports and relevant hospital records (surgery notes, discharge summaries, multidisciplinary team summaries and other correspondence). The EOC were classified into borderline and invasive EOC. We grouped the iEOC into Type I (low‐grade serous, low/moderate‐grade endometrioid, clear cell and mucinous cancers) and Type II [moderate/high‐grade serous, high‐grade endometrioid, undifferentiated, malignant mixed mesodermal (carcinosarcomas)] cancers.^22^, ^23^ Borderline tumours with their excellent survival rates, younger age of onset, differing risk factors and favourable response to surgery were assessed separately from iEOC. Although borderlines were included under Type I in the original proposed model,^16^ the growing consensus has been to exclude them from analysis of Type I and Type II cancers and restrict the focus to iEOC.^23^, ^24^, ^25^

### Symptom ascertainment

Symptom ascertainment has been described previously.^26^ Briefly, symptom data were collected before diagnosis using two methods: a self‐completed questionnaire (Q) and review of primary‐care records (general practitioner records; GP). We used a checklist of ovarian cancer symptoms (pelvic/abdominal pain or discomfort, increase in abdominal size, abdominal bloating, abdominal lump, indigestion, diarrhoea, constipation, nausea/vomiting, irregular vaginal bleeding, urinary frequency or urgency, fatigue, loss of appetite, weight loss, back pain).

Only symptoms that were new within 15 months before diagnosis were included in the analysis (to exclude longstanding symptoms unrelated to the diagnosis of cancer). This cut‐off was the longest interval, such that symptoms in cases were more common than in controls.^27^

### First symptom

To assess whether first symptom(s) differed by tumour type, we grouped symptoms into abdominal (pelvic/abdominal pain or discomfort, increase in abdominal size, abdominal bloating, abdominal lump), gastrointestinal (indigestion, diarrhoea, constipation, change in bowel habit, nausea/vomiting), gynaecological (irregular vaginal bleeding), urinary (frequency or urgency) or systemic (fatigue, weight loss, loss of appetite).

### Time intervals

We followed the Aarhus statement^28^ and used clearly defined key time‐points (see Supplementary material, Appendix S1). We only used questionnaire data to calculate patient intervals because symptom‐onset dates are rarely recorded in primary‐care records. Similarly, primary‐care records were used to calculate diagnostic intervals because they contained the most accurate dates for first presentation.

### First referral

We used medical record data (primary‐care records, hospital letters, multidisciplinary team summaries) to identify the mode and date of first referral. We grouped referrals into (1) 2‐week cancer referral to gynaecological oncology, (2) 2‐week cancer referral to nongynaecological specialties, (3) routine referral to general gynaecology, (4) routine referral to nongynaecological specialties, and (5) accident and emergency (this last group was included because 24% of cancers in the UK are diagnosed via emergency presentations).^29^

### Statistical analysis

We hypothesised that if time to diagnosis was a significant contributor to stage at diagnosis, then time‐to‐diagnosis intervals should be different between the biologically disparate Type I and Type II iEOC. Specifically, we expected longer time to diagnosis to be associated with more advanced stage in Type I iEOC, but not in Type II iEOC (because of aggressive disease/rapid progression). We also reasoned that women with Type II iEOC would be more symptomatic and have different referral patterns compared with Type I iEOC. To examine our hypotheses, we compared time‐to‐diagnosis intervals and first symptom frequency by tumour type: Type I versus Type II iEOC subtypes and for borderline tumours versus all invasive EOC tumours.

We calculated the proportion of women who had patient and diagnostic intervals of 0 to <3, ≥3 to <6, ≥6 to <9 and ≥9 months for each tumour group. We adjusted patient intervals to accommodate for missing symptom dates by multiplying the proportion of women with known symptom dates by the proportion of all women with a symptom. This seemed reasonable because the proportion of women with no calculable interval due to missing onset dates was similar for Type I (77%) and Type II (79%) iEOC.

To assess if first referral differed according to tumour type, for each referral mode and tumour type we compared the proportion of women and the median time from first referral to diagnosis.

The chi‐square test (or Fisher's exact test when expected cell frequency was less than five) was used to assess the relationship between first symptom type and tumour type (Type I versus II iEOC and borderline versus iEOC). A stratified Wilcoxon rank sum test^30^ was used to assess the relationship between tumour type (Type I versus Type II) and categorical time intervals, and between total interval and stage, separately for Type I and Type II tumours. We assessed the relationship between patient/diagnostic intervals and FIGO stage for Type I and Type II cancers separately using the Wilcoxon rank sum test.

All statistical analyses were performed using STATA 12 (StataCorp, College Station, TX, USA). A *P*‐value of <0.05 was considered statistically significant. All statistical tests were two‐sided.

## Results

A total of 227 women with newly diagnosed primary EOC were recruited between 2006 and 2008. Of these women, 194 had invasive EOC and 33 had borderline epithelial ovarian tumours. In all, 222 (98%) completed questionnaires, primary‐care records were received for 199 (88%) women and for 194 (85%) women we had both. Of the iEOC, 60 were Type I (six low‐grade serous, 13 low‐grade endometrioid, 17 clear cell, 19 mucinous, five low grade mixed) and 134 were Type II (98 high‐grade serous, nine high‐grade endometrioid, three undifferentiated, ten carcinosarcoma, one transitional, eight adenocarcinoma, five high‐grade mixed) iEOC.

Table 1 details demographic and clinical details for women with Type I and Type II iEOC and borderline tumours. Women across all tumour groups were similar for age at diagnosis (mean age between 62 and 65 years) and ethnicity (≥ 95% White). Approximately 90% of women with Type II iEOC and borderline tumours were postmenopausal, but only 75% of women with Type I iEOC were postmenopausal. As expected, a higher proportion (78%) of Type I than Type II (21%) had early stage (I/II) disease. Among those with borderline tumours, 73% had early‐stage disease.

**Table 1:** Demographics and clinical details (*n *= 227)

	Type I iEOC (*n* = 60)	Type II iEOC (*n* = 134)	Borderline ovarian tumours (*n* = 33)
**Age at diagnosis, (years)**			
Mean (±SD)	62.2 (10.7)	65.3 (9.8)	63.9 (12.2)
Range	46–83	46–90	45–87
**Ethnicity**			
White	57 (95%)	130 (97%)	33 (100%)
Other	3 (5%)	4 (3%)	0 (0%)
**Menopausal status**			
Unknown	5 (8%)	1 (1%)	–
Perimenopausal	10 (17%)	14 (10%)	3 (9%)
Postmenopausal	45 (75%)	119 (89%)	30 (91%)
Using HRT	9 (20%)	43 (36%)	8 (27%)
Not using HRT	28 (62%)	65 (55%)	21 (70%)
Unknown if using HRT	8 (18%)	11 (9%)	1 (3%)
**FIGO stage**			
I	42 (70%)	14 (10%)	24 (73%)
II	5 (8%)	14 (10%)	0 (0%)
III	11 (18%)	80 (60%)	3 (9%)
IV	2 (3%)	25 (19%)	0 (0%)
Unstaged	–	1 (1%)	6 (18%)
**Tumour grade**			
1	23 (38%)	–	NA
2	18 (30%)	22 (16%)	
3	12 (20%)	91 (68%)	
Unknown	7 (12%)	21 (16%)	

HRT, hormone replacement therapy.

### First symptom

The first symptom reported was similar for Type I and II cancers with the exception of more irregular vaginal bleeding (almost all postmenopausal) in Type I (Q: 15% versus 4%, *P* = 0.01) (see Table 2). Abdominal symptoms were the most common (Q: Type I 77% and Type II 65%), whereas gynaecological symptoms were the least common (Q: Type I 15% and Type II 4%). The reporting of bloating and increased abdominal size was three to four times higher on questionnaire than in primary‐care records (e.g. Type II bloating 40% Q versus 9% GP).

**Table 2:** Nature of first symptom by tumour group

	Questionnaire		GP notes		Questionnaire		GP notes	
	Type I iEOC (*n* = 57)	Type II iEOC (*n* = 132)	Type I iEOC (*n* = 54)	Type II iEOC (*n* = 117)	iEOC (*n* = 189)	Borderline (*n* = 33)	iEOC (*n* = 171)	Borderline (*n* = 28)
**Women with symptom**	*n* = 53	*n* = 124	*n* = 48	*n* = 110	(*n* = 177)	(*n* = 26)	(*n* = 158)	(*n* = 24)
**Abdominal**								
≥1 abdominal first symptom	41 (77%)	80 (65%)	23 (48%)	51 (46%)	121 (68%)	20 (77%)	74 (47%)	10 (42%)
Pelvic/abdominal pain/discomfort	21 (40%)	40 (32%)	14 (29%)	31 (28%)	61 (34%)	8 (31%)	45 (28%)	6 (25%)
Increase in abdominal size	23 (43%)	43 (35%)	8 (17%)	11 (10%)	66 (37%)	11 (42%)	19 (12%)	3 (13%)
Abdomen feels bloated	22 (42%)	49 (40%)	5 (10%)	10 (9%)	71 (40%)	13 (50%)	15 (9%)	0 (0%)
Able to feel a lump in abdomen	5 (9%)	9 (7%)	2 (4%)	6 (5%)	14 (8%)	4 (15%)	8 (5%)	3 (13%)
**Gastrointestinal (GI)**								
≥1 GI first symptom	16 (30%)	42 (34%)	14 (29%)	36 (33%)	58 (33%)	5 (19%)	50 (32%)	5 (21%)
Indigestion	5 (9%)	21 (17%)	4 (8%)	11 (10%)	26 (15%)	3 (12%)	15 (9%)	1 (4%)
Constipation	7 (13%)	19 (15%)	4 (8%)	8 (7%)	26 (15%)	2 (8%)	12 (8%)	2 (8%)
Diarrhoea	2 (4%)	11 (9%)	1 (2%)	4 (4%)	13 (7%)	2 (8%)	5 (3%)	1 (4%)
Change in bowel habit	1 (2%)	1 (1%)	1 (2%)	10 (9%)	2 (1%)	0 (0%)	11 (7%)	2 (8%)
Nausea or vomiting	8 (15%)	8 (6%)	5 (10%)	7 (6%)	16 (9%)	2 (8%)	12 (8%)	0 (0%)
**Gynaecological**								
Irregular vaginal bleeding	8 (15%)	5 (4%)^a^	6 (13%)	4 (4%)^a^	13 (7%)	1 (4%)	10 (6%)	2 (8%)
**Urinary**								
Urinary frequency or urgency	9 (17%)	17 (14%)	8 (17%)	8 (15%)	26 (15%)	8 (31%)	16 (10%)	6 (25%)^a^
**Systemic**								
≥1 systemic first symptom	20 (38%)	54 (44%)	5 (10%)	17 (15%)	74 (42%)	6 (23%)	22 (14%)	0 (0%)^a^
Loss of appetite	6 (11%)	20 (16%)	0 (0%)	5 (5%)	26 (15%)	2 (8%)	5 (3%)	0 (0%)^a^
Weight loss	10 (19%)	17 (14%)	1 (2%)	7 (6%)	27 (15%)	1 (4%)	8 (5%)	0 (0%)^a^
Fatigue	11 (21%)	34 (27%)	4 (4%)	10 (9%)	45 (25%)	4 (15%)	14 (9%)	0 (0%)^a^
**Other**								
Back pain	4 (8%)	13 (10%)	3 (6%)	13 (12%)	17 (10%)	2 (8%)	16 (10%)	2 (8%)

Women may have more than one‐first symptom type. Percentages are calculated with the number of women who reported a (new checklist) symptom on each source as the denominator.

a *P* < 0.05.

Borderline tumours had more urinary frequency/urgency than iEOC (25% versus 10%, *P *= 0.04) recorded as their first symptom in primary‐care records and none had systemic symptoms.

### Time to diagnosis intervals

Patient and diagnostic intervals were similar for Type I and Type II iEOC, although a higher proportion of women with Type I presented within 3 months of symptom onset (Table 3). Total intervals were also comparable: 6.5 months (interquartile range 2.8–10.4) for Type I versus 7.2 months (interquartile range 3.9–12.8) for Type II.

**Table 3:** Patient and diagnostic intervals (months) for Type I and II tumours and according to early versus late stage

	Type I iEOC		Type II iEOC	
Patient interval	(*n* = 57)		(*n* = 132)	
No. with symptom	53		124	
No. with interval	41		98	
0 to < 3 m^a^	29 (70.1%)		64 (51.6%)	
3 to < 6 m^a^	2 (4.8%)		17 (13.7%)	
6 to < 9 m^a^	5 (12.2%)		7 (5.6%)	
≥ 9 m^a^	5 (12.2%)		10 (8.1%)	
**Median months (IQR)**				
Early stage	0.3 (0.3–3.0)	*n* = 33	1.0 (0.3–4.0)	*n* = 18
Late stage	0.3 (0.3–4.5)	*n* = 8	1.8 (0.3–5.0)	*n* = 79

Diagnostic interval	(*n *= 54)		(*n* = 117)	
No. with symptom	48		110	
No. with interval	48		110	
0 to <3 m	23 (47.9%)		44 (40.0%)	
3 to <6 m	10 (20.8%)		29 (26.4%)	
6 to < 9 m	2 (4.2%)		7 (6.4%)	
≥9 m	13 (27.1%)		30 (27.3%)	
**Median months (IQR)**				
Early stage	3.0 (2.0–6.1)	*n* = 37	3.5 (2.2–6.4)	*n* = 24
Late stage	3.7 (1.3–12.8)	*n* = 11	4.1 (1.7–9.8)	*n* = 86

IQR, interquartile range.

a Adjusted by multiplying number of women in interval by proportion of women with symptoms who had an estimable interval.

For both Type I and Type II, median patient intervals were short (within 1 month) and median diagnostic intervals were several months longer than patient intervals (see Supplementary material, Table S1).

In all, 12.2% of women with Type I and 8.1% with Type II cancers delayed presentation for ≥ 9 months (Table 3), and 27% of women with Type I and Type II iEOC had diagnostic intervals of ≥ 9 months.

There were no significant trends between time‐to‐diagnosis intervals and cancer stage in either Type I or Type II tumours (Table 3 and see Supplementary material, Figure S1a,b). Furthermore, 19% (3/16) of women with Type II had early‐stage disease despite long total intervals of at least 9 months and 43% (3/7) of Type I had late‐stage disease despite having short total intervals (see Supplementary material, Table S2). However, all intervals were more likely to be longer in late‐stage than in early‐stage disease.

### Mode of referral

Fourteen women were diagnosed as a result of incidental findings (six Type I, seven Type II, one borderline) and referral pathways were unknown for 11 women. In the remaining women, the most common initial referral across all tumour types was a 2‐week cancer referral to gynaecological oncology (ranging from 34% for Type II to 60% for borderline tumours). A higher proportion of women with Type I versus Type II cancers had a 2‐week cancer referral to gynaecological oncology (*P *= 0.005), whereas more Type II women had a routine referral to nongynaecological specialties (*P *= 0.17) (Table 4). Higher proportion of borderline compared with iEOC had a 2‐week cancer referral to gynaecological oncology (*P *= 0.05); however, numbers were small.

**Table 4:** Median months from first referral to diagnosis by mode of referral and tumour type

Mode of referral	Type I iEOC		Type II iEOC		iEOC		Borderline	
	*n* (%)	Median (IQR) months	*n* (%)	Median (IQR) months	*n* (%)	Median (IQR) months	*n* (%)	Median (IQR) months
2‐week cancer referral to gynaecological oncology	29 (57)^a^	1.5 (1.3–2.0)	41 (34)^a^	1.4 (0.7–1.9)	70 (41)	1.4 (1.1–2.0)	18 (60)	1.5 (1.1, 1.8)
2‐week cancer referral to nongynaecological specialties	5 (10)	2.2 (1.8–2.2)	17 (14)	1.4 (1.2–2.5)	22 (13)	1.4 (1.2–2.5)	2 (7)	3.8 (2.0, 5.5)
Routine to general gynaecology	6 (12)	2.6 (2.1–3.3)	10 (8)	2.7 (2.0–3.7)	16 (9)	2.6 (2.0–3.7)	3 (10)	8.7 (1.6, 11.4)
Routine to nongynaecological specialties^b^	1 (2)	–	22 (18)	2.3 (1.4–5.5)	23 (13)	2.3 (1.4–5.5)	3 (10)	4.6 (2.8, 7.4)
Accident and emergency	10 (20)	1.3 (0.3–2.0)	31 (26)	0.9 (0.5–1.7)	41 (24)	1.0 (0.5–1.7)	4 (13)	1.4 (0.9, 3.1)
**Total referrals**	**51 (100%)**		**121 (100%)**		**172 (100%)**		**30 (100%)**	

iEOC, invasive epithelial ovarian cancer; IQR, interquartile range. Based on medical record data. Women with unknown mode of referral (*n* = 11) or who were diagnosed following an incidental finding (*n* = 14) are excluded.

a *P* < 0.05.

b One woman had a routine referral to clinical oncology as she was still undergoing follow up for colon cancer.

Time from first referral to diagnosis according to referral mode was comparable for Type I versus Type II tumours. Excluding the women who presented as emergencies, time from referral to diagnosis was typically 1 month longer for routine referrals both to general gynaecology and nongynaecological departments. Following referral, women with borderline tumours took longer than women with iEOC to be diagnosed.

## Discussion

### Main findings

To our knowledge, this is the first study to examine time‐to‐diagnosis intervals in iEOC grouped into the molecularly and clinically distinct Type I and Type II iEOC. Despite expected large differences in stage distribution, we found no differences in time‐to‐diagnosis intervals in women with Type I compared with Type II iEOC. Symptom profiles were also broadly comparable for all cancer groups, implying that symptoms may not reflect underlying tumour biology and pathogenesis. An exception to this was that more Type I than Type II cancers had initial symptoms o f irregular vaginal bleeding (a red flag symptom for gynaecological malignancy). However, this was an infrequent symptom. Despite apparently similar symptom profiles, the referral patterns were different among tumour groups. The fact that more women with Type I than Type II iEOC were first referred for a suspected gynaecological malignancy suggests that there are subtle differences in symptom severity, frequency or progression that we were unable to disentangle.

### Strengths and limitations

A major strength of this study is that we used two different sources to collect data on time intervals to ovarian cancer diagnosis – onset of symptoms directly from patients, date of first presentation from primary‐care records and date of diagnosis from secondary care histology records. Using this approach, the high sensitivity of each source for a particular data item was maximised. To truly combine the data into a single interval from symptom onset to diagnosis requires a complex algorithm which we are currently developing. Other strengths include the multicentre design involving ten gynaecological oncology centres across the UK, thereby providing a more representative sample.

It is possible that subtle differences between clear cell and endometrioid tumours have been masked by our Type I/II grouping.^31^ Nevertheless, we feel that within the context of the aims of our study (to estimate time to diagnosis according to a broad dichotomy of aggressive, rapidly advancing cancers versus indolent cancers with favourable prognoses) our chosen grouping would have minimal impact on the outcomes of interest. A gynaecological oncologist reviewed all pathology and related records and confirmed diagnosis. Central pathological review was not undertaken before this analysis. Although there is evidence of discordance in histological subtyping between local and expert review, this relates to reclassification of high‐grade endometrioid, high‐grade mixed and undifferentiated into high‐grade serous.^32^ This was anticipated when we undertook our classification into Type I and Type II disease and therefore does not affect our analysis. Although women were systematically approached, selection bias was inevitable in common with all studies based on individual consent. Another limitation was that patient interval data were based on retrospective report, which can give rise to recall bias and recall error. Although for any systematic errors in symptom reporting (i.e. recall bias), the bias would have been the same for our main comparator groups because women did not know if they had Type I or Type II iEOC at the time of questionnaire completion. In addition, women may not have recalled symptom details accurately because of memory decay (recall error). This could have resulted in overestimation or underestimation of patient intervals. We minimised this by collecting questionnaire data at or before definitive diagnosis. Small numbers in subgroups limited some of our assessments. Finally, six of the women in the current analysis were enrolled in the control (no intervention) arm of the UK Collaborative Trial of Ovarian Cancer Screening (UKCTOCS). It is possible that these women were more health aware but given that the healthy volunteer effect decreases with time from randomisation,^33^ it is of note that recruitment to UKCTOCS was completed in 2005 and the above six women were diagnosed between 2006 and 2008.

### Interpretation

Our patient and diagnostic intervals are comparable to those previously reported (medians between 1 and 4 months).^10^, ^12^, ^14^, ^34^ Also in corroboration of our findings, a large population‐based case–control study in Australia reported no difference in time‐to‐diagnosis intervals for early‐ versus late‐stage iEOC.^6^ We are only aware of one other study^35^ that examined time to diagnosis of EOC by histological subtype. They found that women with serous ovarian cancer had longer mean patient intervals compared with other histological types of epithelial ovarian cancer (12 weeks versus 7 weeks, *P *< 0.05).

The short median patient intervals in our study belie the fact that one in five women with iEOC reported having symptoms for at least 6 months before presenting. Recently, an International Cancer Benchmarking Partnership study identified increased perceived barriers to symptomatic presentation in the UK compared with similar European countries, especially concern about wasting the doctor's time.^36^ Despite the recruitment period being 2006–08, there were considerable delays in diagnosis with one in four women with iEOC (whether Type I or II) experiencing diagnostic intervals of ≥ 9 months. Most women were not referred appropriately, only 41% with iEOC were initially referred to gynaecological oncology. Nevertheless, time from referral to diagnosis was similar for all 2‐week cancer referrals, indicating that suspecting malignancy is more important than diagnosing the correct cancer site. This most likely reflects the reorganisation of cancer services in the National Health Service in the UK, which ensures multidisciplinary review of all new cases in a timely manner with strict standards on time to treatment from primary‐care referral.

If time to diagnosis was a significant contributor to stage at diagnosis, then patient and diagnostic intervals should be different between the biologically disparate Type I and Type II iEOC. Instead it seems that the link between symptoms and tumour biology in ovarian cancer is complex and that a symptom‐based intervention may not result in earlier stage diagnosis of Type II iEOC.

The fact that upper quartiles for late stage intervals tended to be longer than for early stage indicated a bimodal distribution for late stage: either short because of very aggressive disease or long leading to advanced disease. Our findings are in keeping with emerging data on the natural history of high‐grade serous iEOC where modelling suggests that early in the course of disease at a median diameter of about 3 cm, serous ovarian cancers progress to an advanced stage (stage III or IV).^37^ This suggests that in Type II iEOC a symptom‐based intervention is more likely to influence tumour volume than impact on stage. This is supported by the recent prospective study (DOvE study; Diagnosing Ovarian Cancer Early)^38^ where CA125 testing and transvaginal ultrasound in women with ovarian cancer symptoms resulted in detection of lower volume disease in women with high‐grade serous ovarian cancers.^39^ Diagnosis at lower tumour volumes is likely to improve the surgeon's ability to achieve zero residual disease at surgery, a key prognostic factor in disease survival.^40^ In addition, less intra‐tumour heterogeneity may lead to a better response to chemotherapy.

The drive by service providers and patients to raise symptom awareness and introduce systematic CA125 and ultrasound testing in primary care is understandable given the continued significant delays in diagnosis. In our study, one in four women had diagnostic intervals >9 months. However, given the prevalence and non‐specific nature of symptoms^41^ the significant resource implications and potential harm to symptomatic women without ovarian cancer,^42^ it remains controversial as to whether or not current evidence is adequate to support practice change or a prospective randomised controlled trial of a symptom‐based intervention is needed to comprehensively assess impact.

## Conclusions

Overall, shorter diagnostic delays were seen when a cancer was suspected, even if the primary tumour site was not recognised to be ovarian. Despite differences in tumour biology, carcinogenesis and stage at diagnosis, no difference in time to diagnosis for Type I and II ovarian cancers was noted. However, the pattern of referral was different, implying that there may be subtle differences in symptoms. If symptom‐based interventions impact on survival, it is unlikely to be through stage shift. Further research focused on histological subtypes is needed.

## Disclosure of interests

Full disclosure of interests available to view online as supporting information.

## Contribution to authorship

All authors participated in the editing of this manuscript and approved the final version for publication. AWL, PS and UM jointly planned and designed the study. AWL collected and analysed the study data, wrote the first draft of the manuscript and the manuscript revisions. AGM coordinated the study. NB recruited the study participants in London. DM provided statistical support for the analysis. PS and UM supervised the interpretation of the analysis, and revised the manuscript. AWL, PS and UM had full access to all of the data in the study and take responsibility for the integrity of the data and the accuracy of the data analysis as well as the final decision to submit for publication. MW confirmed histological diagnosis. IJ contributed to the main study design and reviewed the final manuscript. All authors accept responsibility for the paper as published.

## Details of ethics approval

Ethics approval was obtained from the Joint University College London/University College Hospital London Research Ethics Committee (05/Q0505/58) on 29 July 2005.

## Funding

This study was funded by Cancer Research UK (C8162/A6138 to PS and AWL and C8162/A10406 to PS and DM); Eve Appeal/Oak Foundation (to UM and IJ) and was supported by researchers at the National Institute for Health Research University College London Hospitals Biomedical Research Centre.

## Supporting information


**Figure S1.** (a) Time from first symptom to presentation by stage at diagnosis and by tumour type—cumulative proportion of women.

**Table S1**. Time to diagnosis intervals (months) by tumour type.

**Table S2.** Total intervals (months) for early versus late stage Type I and Type II tumours.

**Appendix S1**. Supplementary methods.
