# Supplementary material for: Time to diagnosis of Type I or II invasive epithelial ovarian cancers: a multicentre observational study using patient questionnaire and primary care records
Source: BJOG. 2015 May 29;123(6):1012–20. doi: 10.1111/1471-0528.13447 (PMC4855631; doi:10.1111/1471-0528.13447)

Figure S1a Time from first symptom to presentation by stage at diagnosis and by tumour type – cumulative proportion of women

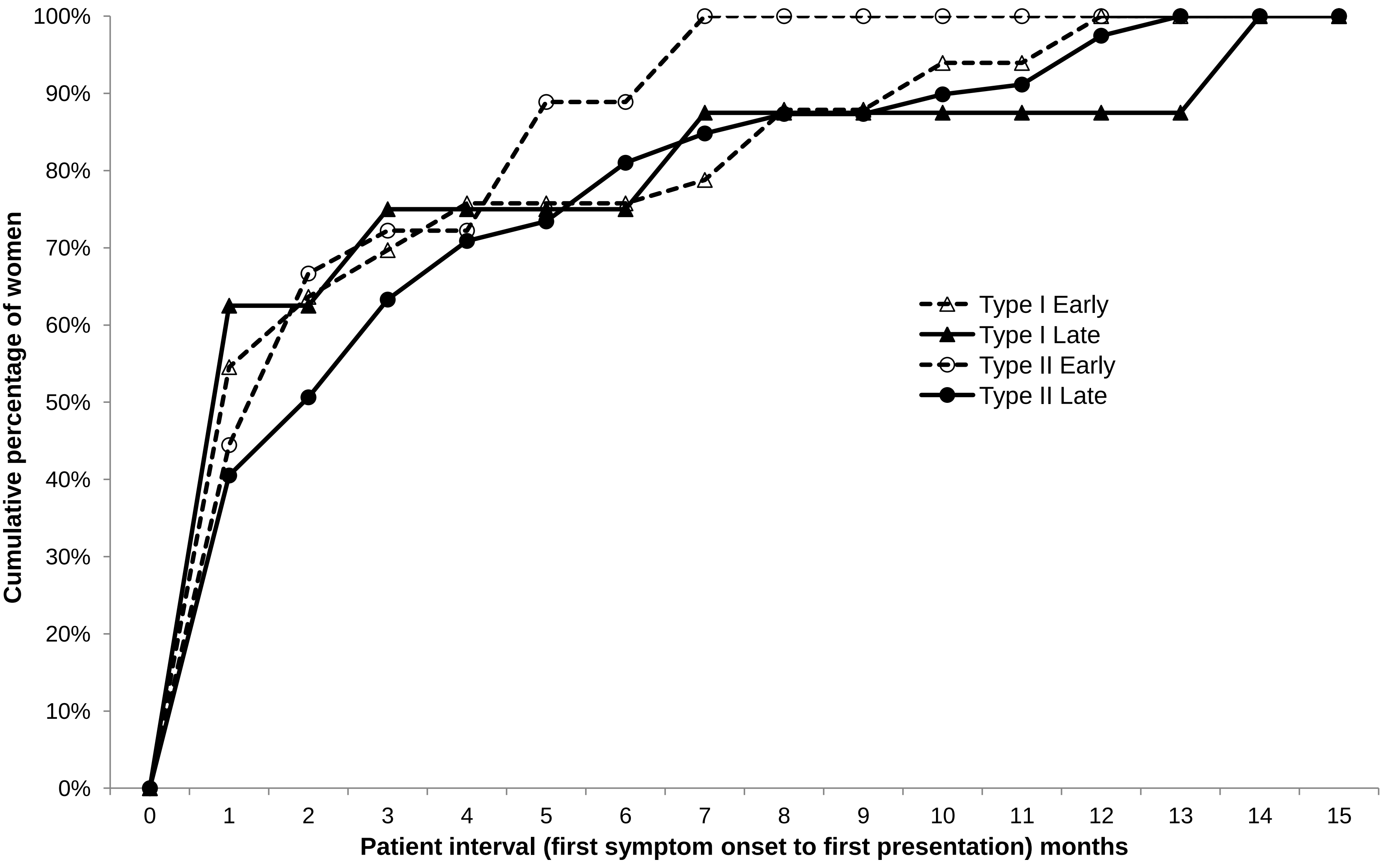

Figure S1b Time from presentation to diagnosis by stage at diagnosis and by tumour type – cumulative proportion of women

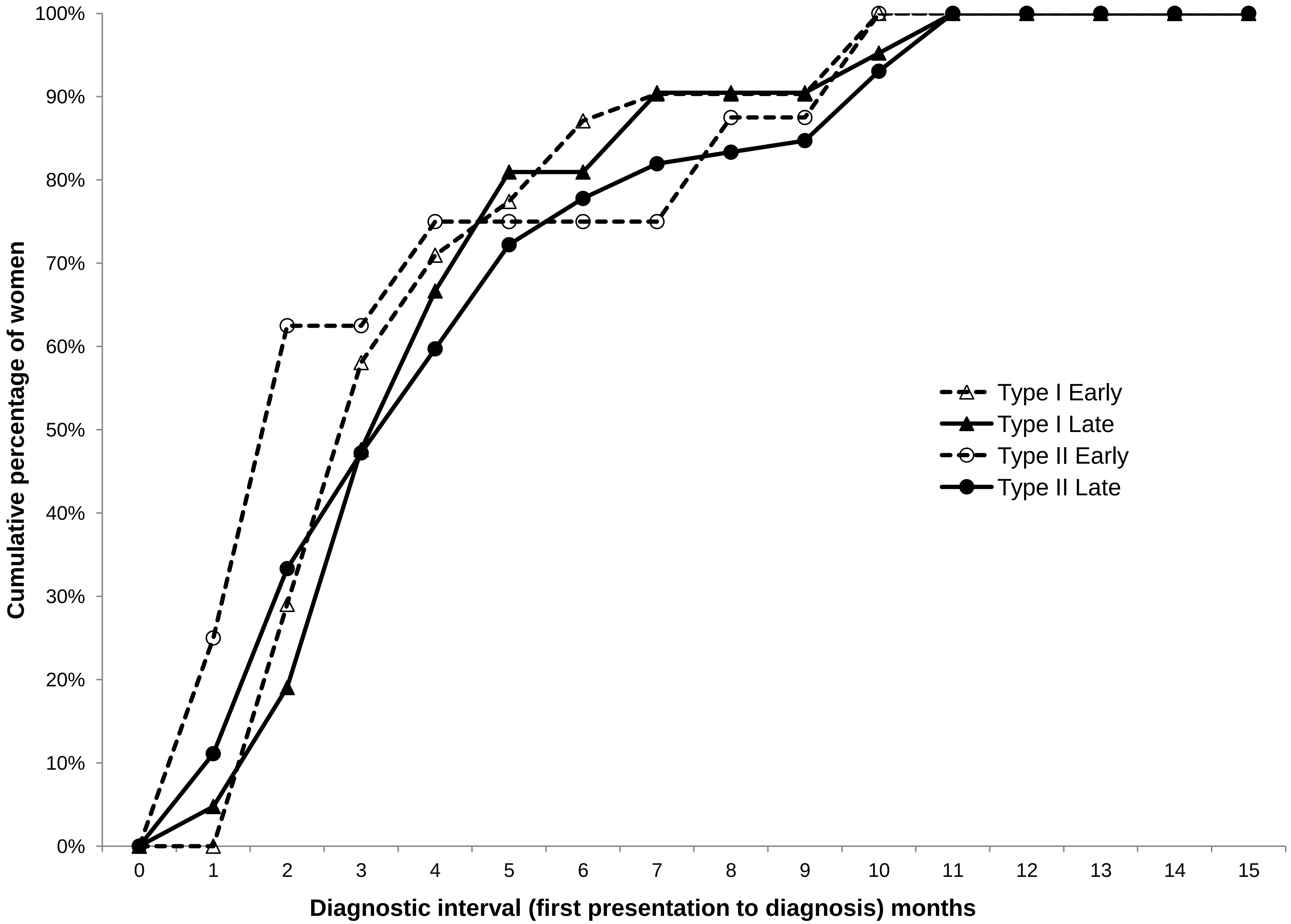

Supplement: Supplementary file 1 — Figure S1. (a) Time from first symptom to presentation by stage at diagnosis and by tumour type—cumulative proportion of women. [file BJO-123-1012-s001.pdf]
