# Supplementary material for: Time to diagnosis of Type I or II invasive epithelial ovarian cancers: a multicentre observational study using patient questionnaire and primary care records
Source: BJOG. 2015 May 29;123(6):1012–20. doi: 10.1111/1471-0528.13447 (PMC4855631; doi:10.1111/1471-0528.13447)
Supplement: Supplementary file 2 — Table S1. Time to diagnosis intervals (months) by tumour type. [file BJO-123-1012-s002.pdf]

Table S1. Time to diagnosis intervals (months) by tumour type

	Median (IQR) months	No. with interval
**Patient Interval**		
Type I	0.3 (0.3, 3.0)	41
Type II	1.0 (0.3, 4.9)	98
iEOC	1.0 (0.3, 4.9)	139
Borderline	3.0 (0.3, 5.9)	21
**Diagnostic Interval**		
Type I	3.3 (1.9, 9.3)	48
Type II	4.0 (1.8, 9.2)	110
iEOC	3.6 (1.9, 9.2)	158
Borderline	4.5 (1.6, 10.5)	24

Note: Patient interval is derived from questionnaire data, diagnostic interval is derived from primary care record data. iEOC=invasive epithelial ovarian cancer, IQR=interquartile range.
