# Supplementary material for: Time to diagnosis of Type I or II invasive epithelial ovarian cancers: a multicentre observational study using patient questionnaire and primary care records
Source: BJOG. 2015 May 29;123(6):1012–20. doi: 10.1111/1471-0528.13447 (PMC4855631; doi:10.1111/1471-0528.13447)
Supplement: Supplementary file 3 — Table S2. Total intervals (months) for early versus late stage Type I and Type II tumours. [file BJO-123-1012-s003.pdf]

**Table S2.** Total intervals (months) for early versus late stage Type I and II tumours

| | Total interval | | | | | | | | | |
|---|---|---|---|---|---|---|---|---|---|---|
| | **Type I n=32** | | | | | **Type II n=77** | | | | |
| | 0-<3m | 3-<6m | 6-<9m | ≥9m | No. with interval | 0-<3m | 3-<6m | 6-<9m | ≥9m | No. with interval |
| Early stage | 7 (28%) | 4 (16%) | 3 (12%) | 11 (44%) | 25 | 3 (19%) | 4 (25%) | 6 (38%) | 3 (19%) | 16 |
| Late stage | 3* (43%) | 0 (0%) | 0 (0%) | 4 (57%) | 7 | 11** (18%) | 13 (21%) | 7 (11%) | 30 (49%) | 61 |

Excludes women with missing stage or time to diagnosis intervals.
*1 presented as an emergency, **3 presented as an emergency
