# Supplementary material for: Time to diagnosis of Type I or II invasive epithelial ovarian cancers: a multicentre observational study using patient questionnaire and primary care records
Source: BJOG. 2015 May 29;123(6):1012–20. doi: 10.1111/1471-0528.13447 (PMC4855631; doi:10.1111/1471-0528.13447)
Supplement: Supplementary file 4 — Appendix S1. Supplementary methods. [file BJO-123-1012-s004.pdf]

Appendix S1. Definitions for key time points and time to diagnosis intervals

**Time point or interval**	**Definition**
First symptom onset	Onset date of the earliest symptom (new in the 15 months before diagnosis)
First presentation	First symptomatic presentation to primary care (or accident and emergency if not seen in primary care)
First referral	First referral to secondary care to manage symptoms (even if this did not lead to diagnosis)
Date of diagnosis	Date of histological confirmation of diagnosis either at primary surgery or biopsy/cytology (whichever was earliest)

Time points on the questionnaire were collected as month and year - a midpoint (typically the 15th of the month) was used for analysis unless a specific day was provided. Whenever first symptom and first presentation occurred in the same month (with no dates specified) we defined patient interval to be ten days (first presentation was unlikely to be on the same day as symptom onset).
